# Regulation of Kv2.1 channel inactivation by phosphatidylinositol 4,5-bisphosphate

**DOI:** 10.1038/s41598-018-20280-w

**Published:** 2018-01-29

**Authors:** Mayra Delgado-Ramírez, José J. De Jesús-Pérez, Iván A. Aréchiga-Figueroa, Jorge Arreola, Scott K. Adney, Carlos A. Villalba-Galea, Diomedes E. Logothetis, Aldo A. Rodríguez-Menchaca

**Affiliations:** 1 Departamento de Fisiología y Biofísica, Facultad de Medicina, Universidad Autónoma de San Luis Potosí, San Luis Potosí, SLP 78210 Mexico; 2 Instituto de Física, Universidad Autónoma de San Luis Potosí, Universidad Autónoma de San Luis Potosí, San Luis Potosí, SLP 78290 Mexico; 3 CONACYT, Facultad de Medicina, Universidad Autónoma de San Luis Potosí, San Luis Potosí, SLP 78210 Mexico; 4 Department of Physiology and Biophysics, Virginia Commonwealth University School of Medicine, Richmond, VA 23298 USA; 5 Department of Physiology and Pharmacology, Thomas J. Long School of Pharmacy & Health Sciences, University of the Pacific, Stockton, CA 95211 USA; 6 Department of Pharmaceutical Sciences, School of Pharmacy, Bouvé College of Health Sciences, Northeastern University, Boston, MA 02115 USA; 7 Present Address: Department of Neurology, Northwestern University, Chicago, IL 60611 USA

## Abstract

Phosphatidylinositol 4,5-bisphosphate (PIP_2_) is a membrane phospholipid that regulates the function of multiple ion channels, including some members of the voltage-gated potassium (Kv) channel superfamily. The PIP_2_ sensitivity of Kv channels is well established for all five members of the Kv7 family and for Kv1.2 channels; however, regulation of other Kv channels by PIP_2_ remains unclear. Here, we investigate the effects of PIP_2_ on Kv2.1 channels by applying exogenous PIP_2_ to the cytoplasmic face of excised membrane patches, activating muscarinic receptors (M1R), or depleting endogenous PIP_2_ using a rapamycin-translocated 5-phosphatase (FKBP-Inp54p). Exogenous PIP_2_ rescued Kv2.1 channels from rundown and partially prevented the shift in the voltage-dependence of inactivation observed in inside-out patch recordings. Native PIP_2_ depletion by the recruitment of FKBP-Insp54P or M1R activation in whole-cell experiments, induced a shift in the voltage-dependence of inactivation, an acceleration of the closed-state inactivation, and a delayed recovery of channels from inactivation. No significant effects were observed on the activation mechanism by any of these treatments. Our data can be modeled by a 13-state allosteric model that takes into account that PIP_2_ depletion facilitates inactivation of Kv2.1. We propose that PIP_2_ regulates Kv2.1 channels by interfering with the inactivation mechanism.

## Introduction

Voltage-gated potassium (Kv) channels are integral membrane proteins that enable the passage of potassium ions (K^+^) across cell membranes. They open and close in response to changes in transmembrane voltage, and are involved in numerous physiological processes, for example, in the generation of action potentials^1^.

Almost all Kv channels share a similar mechanism of operation. Upon depolarization, Kv channels transition from a resting (closed) to an activated (open) state, however, during prolonged depolarizations, Kv channels switch to an inactivated (open non-conductive) state^2^. Transitions between these three states (gating) of Kv channels can be modulated by different stimuli. For instance, phosphorylation^3^, SUMOylation^4,5^ polyunsaturated fatty acids^6^, accessory subunits^7^ and different natural and synthetic compounds^8,9^, are well known modulators of Kv channel gating. Phosphoinositides, particularly phosphatidylinositol 4,5-bisphosphate (PIP_2_), have also been shown to modulate the gating mechanism of several Kv channels^10–12^, although, some of these results have been debated^13,14^.

PIP_2_ is a minor phospholipid found in the inner leaflet of the plasma membrane and plays an important role in modulating several ion channels^16^. While it is well established that some members of the Kv channel family, specifically Kv1.2 and Kv7, are regulated by PIP_2_^11,16^, studies in other Kv channels have shown contradictory results. Oliver *et al*. (2004) first reported the modulation of Kv1.1/Kβ1.1, Kv1.4 and Kv3.4 channels by PIP_2_. Application of PIP_2_ micelles to the cytoplasmic side of membrane patches abolished the fast (N-type) inactivation of these channels^10^. However, Kruse *et al*.^13^ using whole-cell recordings and enzymatic methods to deplete native PIP_2_ in intact cells, did not report PIP_2_ sensitivity for several Kv channels, including channels previously reported to be PIP_2_ sensitive (Kv1.1/Kβ1.1, Kv1.4 and Kv3.4). Kv2.1 is another ion channel that was first reported to be PIP_2_ sensitive. Hilgemann *et al*.^17^ reported that Kv2.1 channels were rescued from rundown in excised inside-out patches by applying exogenous PIP_2_. However, experiments using intact cells failed to demonstrate the PIP_2_ sensitivity of Kv2.1^13^. The reasons why some Kv channels (e.g. Kv2.1) show PIP_2_ sensitivity in excised patches, but not in intact cells still remains poorly understood and deserves further investigation.

In this work, we explored the PIP_2_ regulation of Kv2.1 channels expressed in HEK293 cells by employing different strategies to manipulate PIP_2_ levels in excised patches and intact cells. In excised inside-out patches, PIP_2_ recovered the rundown of Kv2.1 channels and prevented the shift in the voltage dependence of inactivation. In whole-cell experiments, native PIP_2_ depletion by membrane translocatable enzymes or M1R activation facilitated the inactivation of Kv2.1 channels. Our data and quantitative analysis suggest that PIP_2_ depletion increases the transitions from closed- and open-states to the inactivated-states.

## Results

### PIP_2_ regulates Kv2.1 channels activity in excised inside-out patches

**Figure 1:**
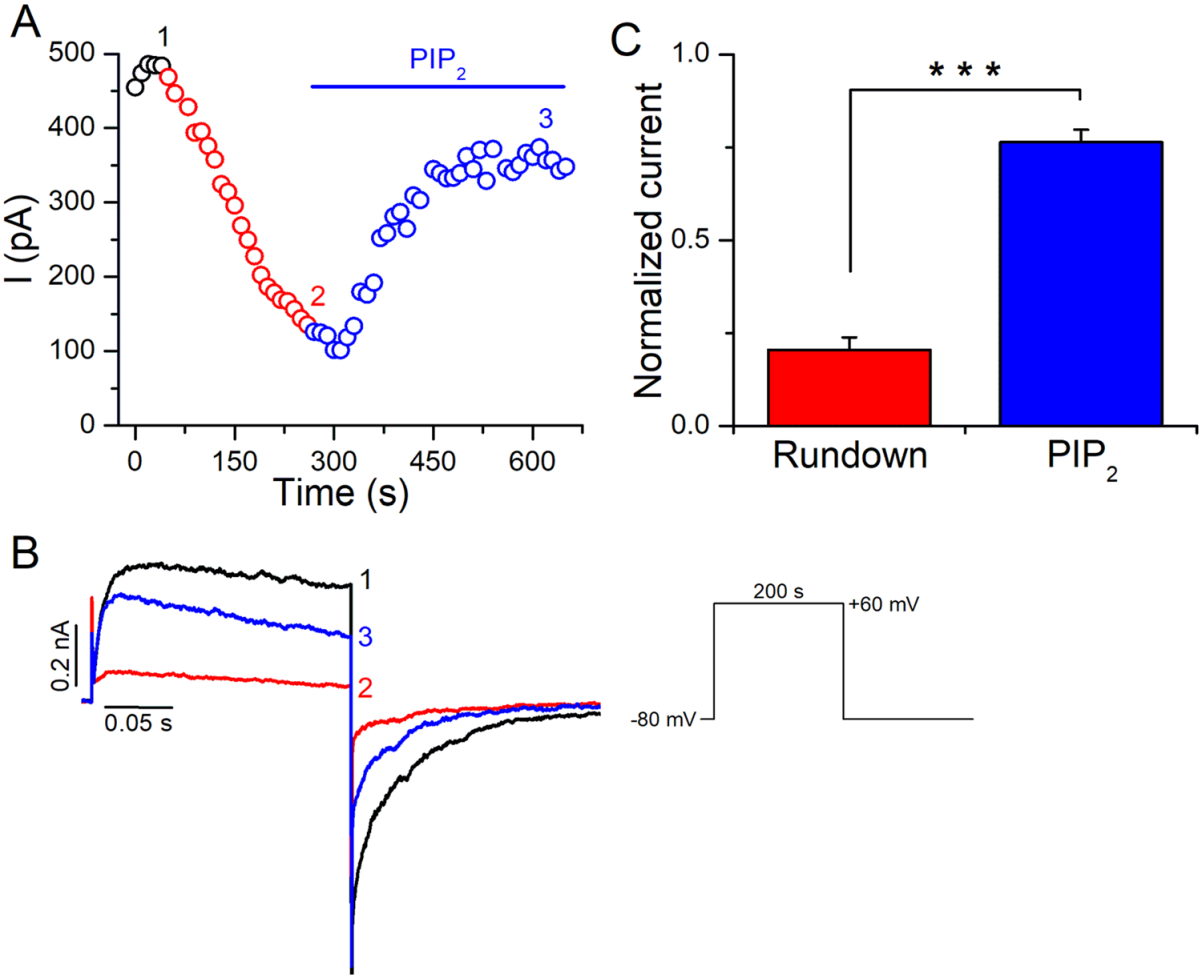
Effect of exogenous PIP_2_ on the activity of Kv2.1 channels. (**A**) Representative temporal course of the current amplitude at +60 mV before (black) and during (red) current rundown and during 10 μM PIP_2_ (blue) application. (**B**) Representative traces recorded with the protocol shown *inset* at the time points indicated by the numbers in (**A**). (**C**) Average normalized current amplitudes after rundown and exposure to 10 μM PIP_2_ (n = 11, ***p < 0.001). Error bars represent ±SEM.

**Figure 2:**
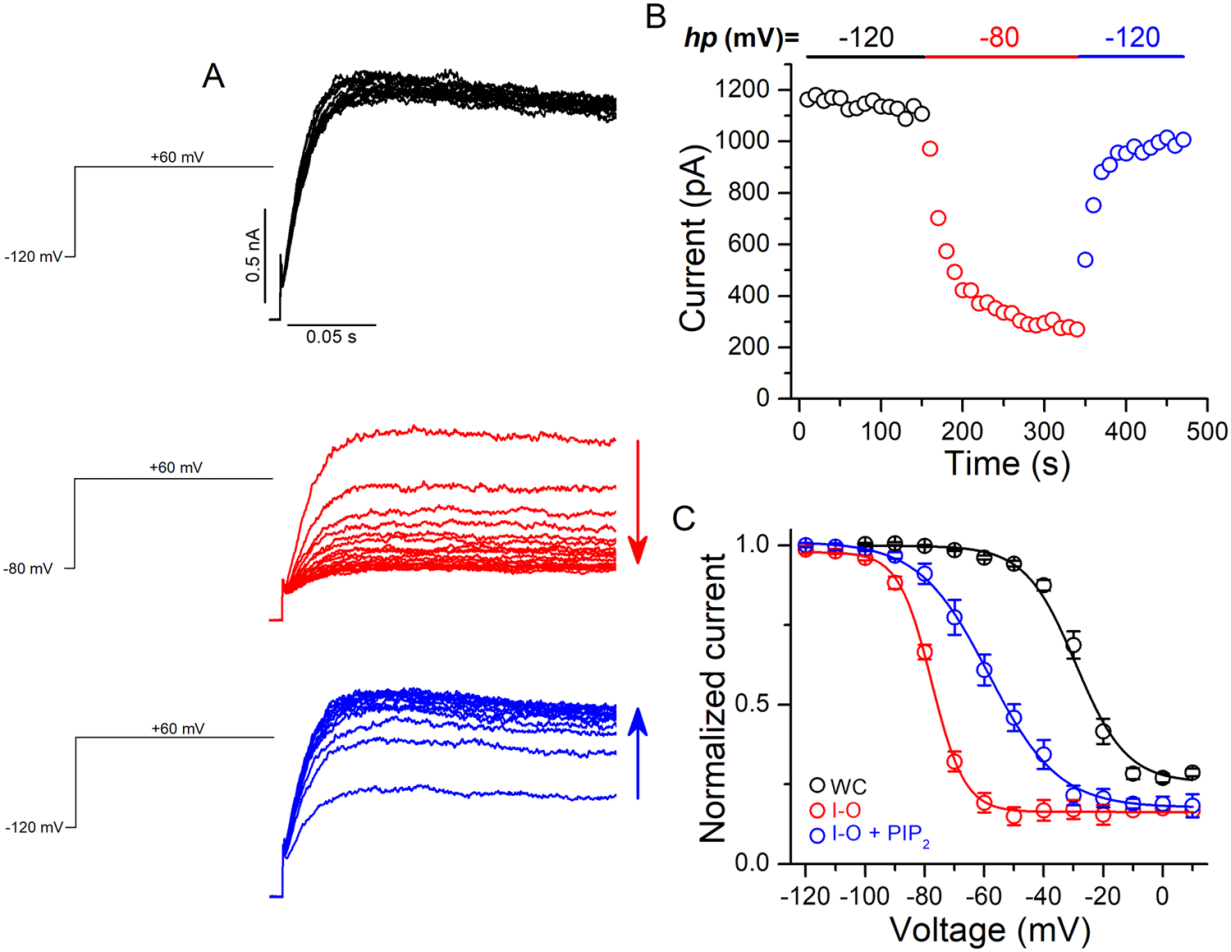
Effect of holding potential (hp) on Kv2.1 current rundown in excised inside-out patches. (**A**) Kv2.1 current traces recorded with protocols shown on left. (**B**) Temporal course of Kv2.1 currents after the change of hp from −120 to −80 mV and then back to −120 mV. (**C**) Voltage-dependence of inactivation of Kv2.1 channels determined under whole-cell (black), inside-out (red), and inside-out in presence of 10 μM PIP_2_ (blue). Data points are mean ± SEM (n = 7–10).

Patch excision leads to a gradual loss of PIP_2_ from the plasma membrane, and alterations of channel function that accompany patch excision can reflect PIP_2_ dependence. For instance, rundown and changes in the voltage-dependence of Kv1.2 channels in inside-out patches can be reduced by manipulations that slow the loss of PIP_2_ and can be reversed by application of exogenous PIP_2_ to the intracellular face of the patch^11^. To test PIP_2_ dependence on Kv2.1 channels, we first recorded Kv2.1 currents in excised inside-out patches. Following patch excision into symmetrical K^+^, Kv2.1 currents activated by a test pulse to + 60 mV from a holding potential of −80 mV, successively decreased in amplitude (rundown) to ~20% of the initial current (Fig. 1A–C). Bath application of exogenous PIP_2_ (10 μM) to the excised inside-out patch significantly reversed the current rundown to ~76% of the initial current (Fig. 1A–C), while doubling the PIP_2_ concentration did not further reverse the current rundown (Supplementary Fig. S1). Similarly, application of 2 mM Mg-ATP to induce the phosphorylation of PIP by the lipid kinases associated to the patch, partially recovered the Kv2.1 currents from rundown (Supplementary Fig. S2). Additionally, the use of a phosphatase inhibitor-containing solution (FVPP^18^), to limit the known PIP_2_ dephosphorylation in inside-out patches slowed the rundown of Kv2.1 currents; the rundown rate (τ_rundown_) in normal external solution was 105.7 ± 9.2 s compared to 298.4 ± 39.8 s in FVPP (*p* < 0.01, Supplementary Fig. S3). A striking observation in inside-out patches was that recordings of Kv2.1 currents using a more negative holding potential (−120 mV) did not show a significant rundown (Fig. 2A,B). However, after switching the holding potential to −80 mV, Kv2.1 currents quickly randown, but could be recovered by returning the holding potential back to −120 mV (Fig. 2A,B). It is known that voltage-dependent gating can be modulated in inside-out patches^11,12,19^. We hypothesized that in inside-out patches the voltage-dependence of inactivation could be shifted to hyperpolarizing potentials and consequently, at our holding potential of −80 mV Kv2.1 currents could be inactivating, as this channel shows preferential closed-state inactivation^20^. To test this hypothesis, we compared the inactivation curves of Kv2.1 channels recorded in the whole-cell and inside-out configurations. Under whole-cell conditions, the V_1/2_ of inactivation was −28.3 ± 1.4 mV (*k* = 5.6 ± 0.3 mV), whereas it was −76.9 ± 1.6 mV (*k* = 5.3 ± 0.3 mV) in the inside-out patch configuration (Fig. 2C). Thus in the inside-out patch mode, the inactivation curve was largely shifted to hyperpolarized potentials (ΔV_1/2_ = 48.6 mV). From the curve in the inside-out patch mode it can be seen that there is significant inactivation at −80 mV, in contrast to when the patch is held at −120 mV. Thus, it is likely that the rundown observed in the inside-out patch recordings at a holding potential of −80 mV was due to an accumulation of inactivation of Kv2.1 channels, as rundown was not observed at a holding potential of −120 mV, where inactivation does not take place. Because PIP_2_ could recover the current from rundown, we tested if this effect was related to the inactivation of Kv2.1. As anticipated, application of PIP_2_ partially prevented the hyperpolarizing shift in the inactivation curve of Kv2.1 channels (V_1/2_ = −57.8 ± 1.3 mV, *k* = 10.9 ± 1.0 mV, Fig. 2C). This partial effect of PIP_2_ suggests that there are additional factors contributing to the difference between the whole-cell and inside-out patch inactivation curves. Altogether, these results suggest that the Kv2.1 rundown observed in inside-out patches is due to an accumulation of inactivation at −80 mV (the holding potential), as the voltage dependence of inactivation is shifted to hyperpolarized potentials. The recovery of Kv2.1 from rundown induced by PIP_2_ is in turn due to a shift of the inactivation curve towards more depolarized potentials.

### Effects of native PIP_2_ depletion on Kv2.1 channels

Several ion channels reported to be PIP_2_ dependent by means of their responses to exogenous PIP_2_ application in excised inside-out patches, were shown to be insensitive to PIP_2_ depletion in intact cells^13^. A number of reasons have been proposed to explain these disparities; among them is the incorporation of large amounts of PIP_2_ to the inner leaflet of the plasma membrane (in inside-out patches), generating unphysiological protein conformations^14^. Therefore, we turned to whole-cell recordings to study modulation of Kv2.1 channels following native PIP_2_ depletion in intact cells. For this purpose, we expressed Kv2.1 channels together with a rapamycin-translocatable lipid 5-phosphatase (FKBP-Inp54p) and the membrane anchor LDR-CFP. The FKBP and LDR domains dimerize when rapamycin is added to cells, leading to the dephosphorylation of PIP_2_ on the 5′ position to PI4P^21^. In all experiments, we used 1 μM rapamycin. We also expressed Kv2.1 channels together with the M1 muscarinic receptor (M1R). M1R is a Gq-coupled receptor that activates phospholipase C (PLC) leading to PIP_2_ depletion^22^. In all experiments, we used 10 μM acetylcholine (ACh) to activate the M1R.

**Figure 3:**
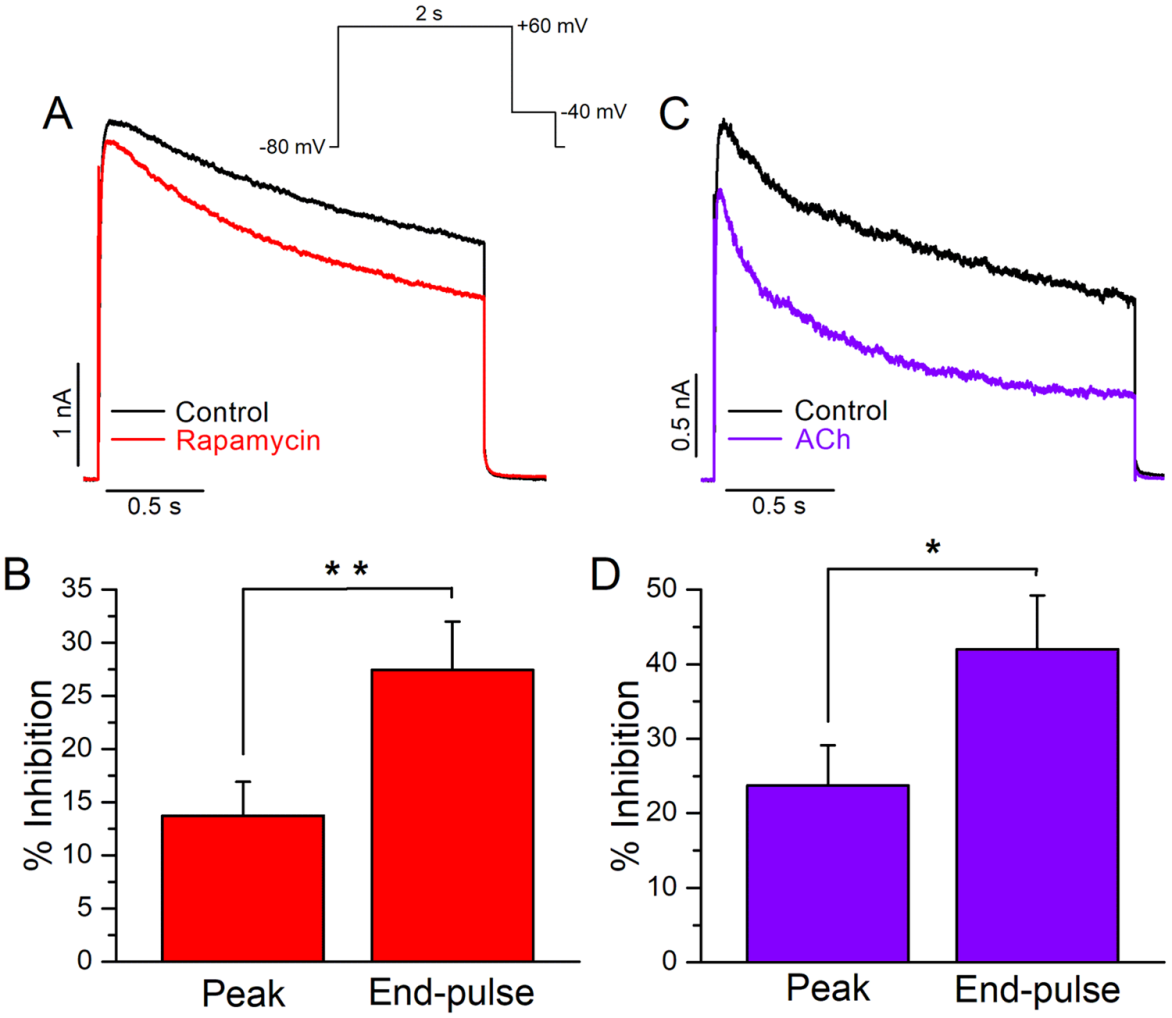
Impact of PIP_2_ depletion on Kv2.1 currents. (**A**) Representative current traces recorded at + 60 mV from a holding potential (hp) of −80 mV under whole-cell configuration. Traces are shown before (control) and after application of 1 μM rapamycin. (**B**) Average peak and end-pulse current inhibition by rapamycin (n = 10). (**C**) Representative Kv2.1 current traces recorded before (control) and after application of 10 μM acetylcholine. (**D**) Average peak and end-pulse current inhibition by acetylcholine (n = 8). Error bars represent ± SEM.

Recently, Kv2.1 channels were reported to be insensitive to PIP_2_ depletion by several experimental approaches^13^. In this work, the amplitude of Kv2.1 currents was barely affected by PIP_2_ depletion; however, no effects on other Kv2.1 biophysical parameters were studied. In our hands, we observed that the recruitment of the 5-phosphatase (FKBP-Inp54p) to the plasma membrane by rapamycin had small but significant effects on the amplitude of Kv2.1 currents (Fig. 3A). The peak- and end-pulse currents were inhibited 13.7 ± 3.1% (*p* < 0.01) and 27.4 ± 4.5% (*p* < 0.01; Fig. 3B), respectively. The larger inhibition at the end of the test pulse is likely due to an acceleration of inactivation (τ_control_ = 1.2 ± 0.2 s and τ_rapamycin_ = 0.77 ± 0.16 s). We also expressed Kv2.1 channels together with M1R. Upon activation of M1R, Kv2.1 peak- and end-pulse currents were inhibited 23.7 ± 5.3% (*p* < 0.01) and 42.1 ± 7.2% (*p* < 0.01; Fig. 3C,D). This larger reduction upon M1R activation could be related to loss of PIP_2_ and to effects of products generated downstream of PIP_2_ hydrolysis induced by PLC activation, as was previously suggested^13^. The inactivation rate was also accelerated after M1R activation (τ_control_ = 0.94 ± 0.16 s and τ_M1R_ = 0.68 ± 0.07 s).

From these results, we concluded that PIP_2_ depletion in intact cells shows smaller effects on the ion conduction of Kv2.1 channels than in excised patches. Thus, we next turned to study the effects of PIP_2_ depletion on the voltage-dependent gating of Kv2.1 channels.

### The voltage-dependence of activation of Kv2.1 channels is not altered by PIP_2_ depletion

**Figure 4:**
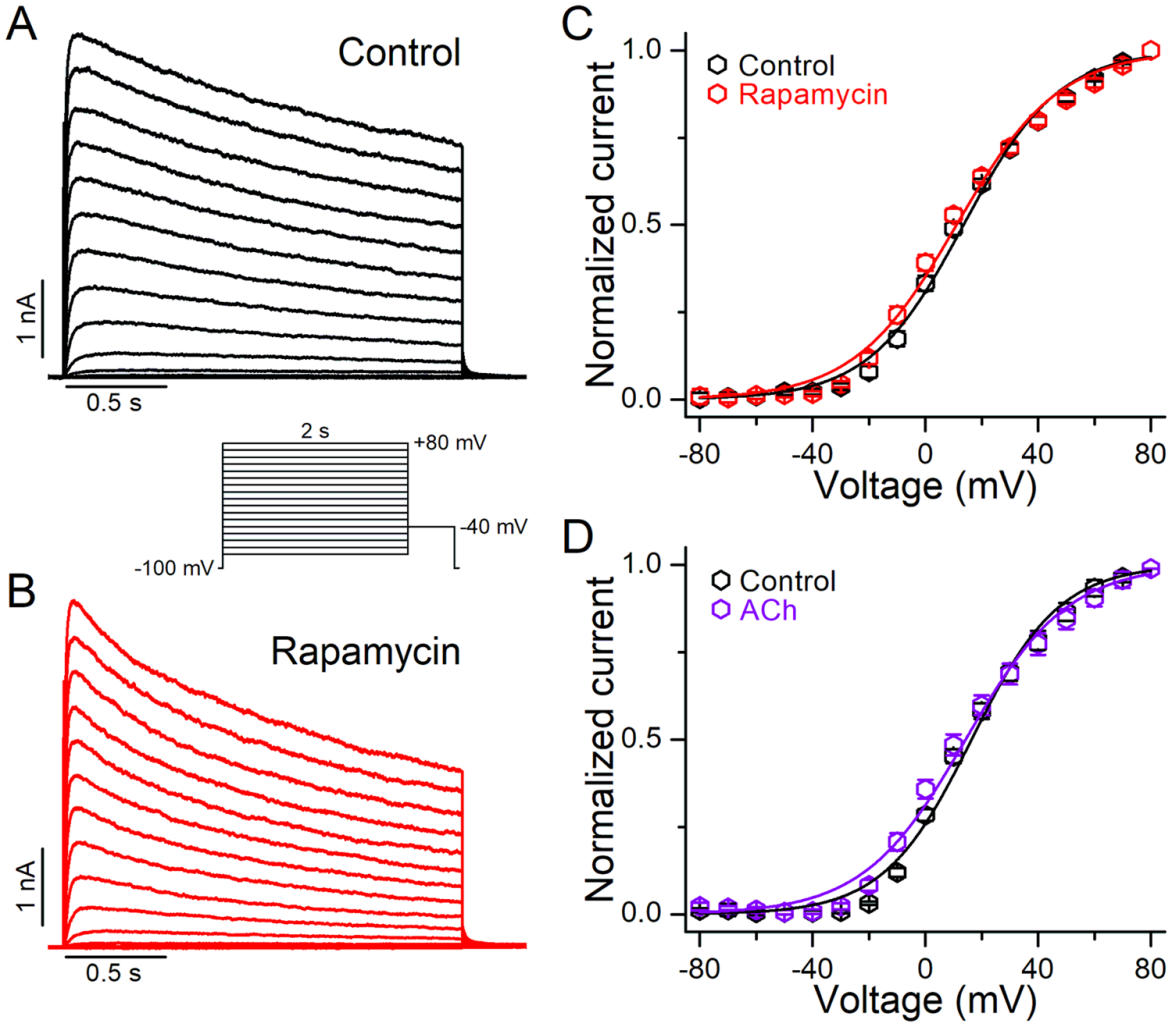
Effect of PIP_2_ depletion on the voltage-dependence of activation of Kv2.1 channels. (**A,B**) Representative Kv2.1 currents traces obtained in response to an activation protocol (*inset*) before (**A**) and after application of 1 μM rapamycin (**B**). (**C**) Activation curves of Kv2.1 channels determined in control conditions (black) and after cells were perfused with 1 μM rapamycin (red, n = 10). (**D**) Activation curves of Kv2.1 channels determined in control conditions (black) and after cells were perfused with 10 μM acetylcholine (violet, n = 8). Data points are mean ± SEM.

To examine the effect of PIP_2_ depletion on the voltage-dependence of activation of Kv2.1 channels, we determined the conductance-voltage relations (G-V curves) recorded from cells studied in the whole-cell mode before (control) and after PIP_2_ depletion induced by the application of rapamycin (Fig. 4). Figure 4A,B shows representative currents traces before (control, A) and after application of rapamycin (B), recorded with the protocol shown in the inset. The midpoint voltage of activation (V_1/2_) was 13.8 ± 0.9 mV (*k* = 15.8 ± 0.6 mV) under control conditions, and was not significantly modified after rapamycin application (V_1/2_ = 11.5 ± 1.5 mV, *k* = 17.3 ± 0.9 mV, *p* > 0.05) (Fig. 4C). Similarly, activation of M1R did not induce a significant change in the V_1/2_ of activation (Fig. 4D, Supplementary Fig. S4). The V_1/2_ was 15.2 ± 1 mV (*k* = 16.9 ± 0.9 mV) under control conditions and 12.8 ± 2.2 mV (*k* = 17.9 ± 1.3 mV) after M1R activation (*p* > 0.05).

### PIP_2_ depletion induced a hyperpolarizing shift in the voltage-dependence of inactivation of Kv2.1 channels

**Figure 5:**
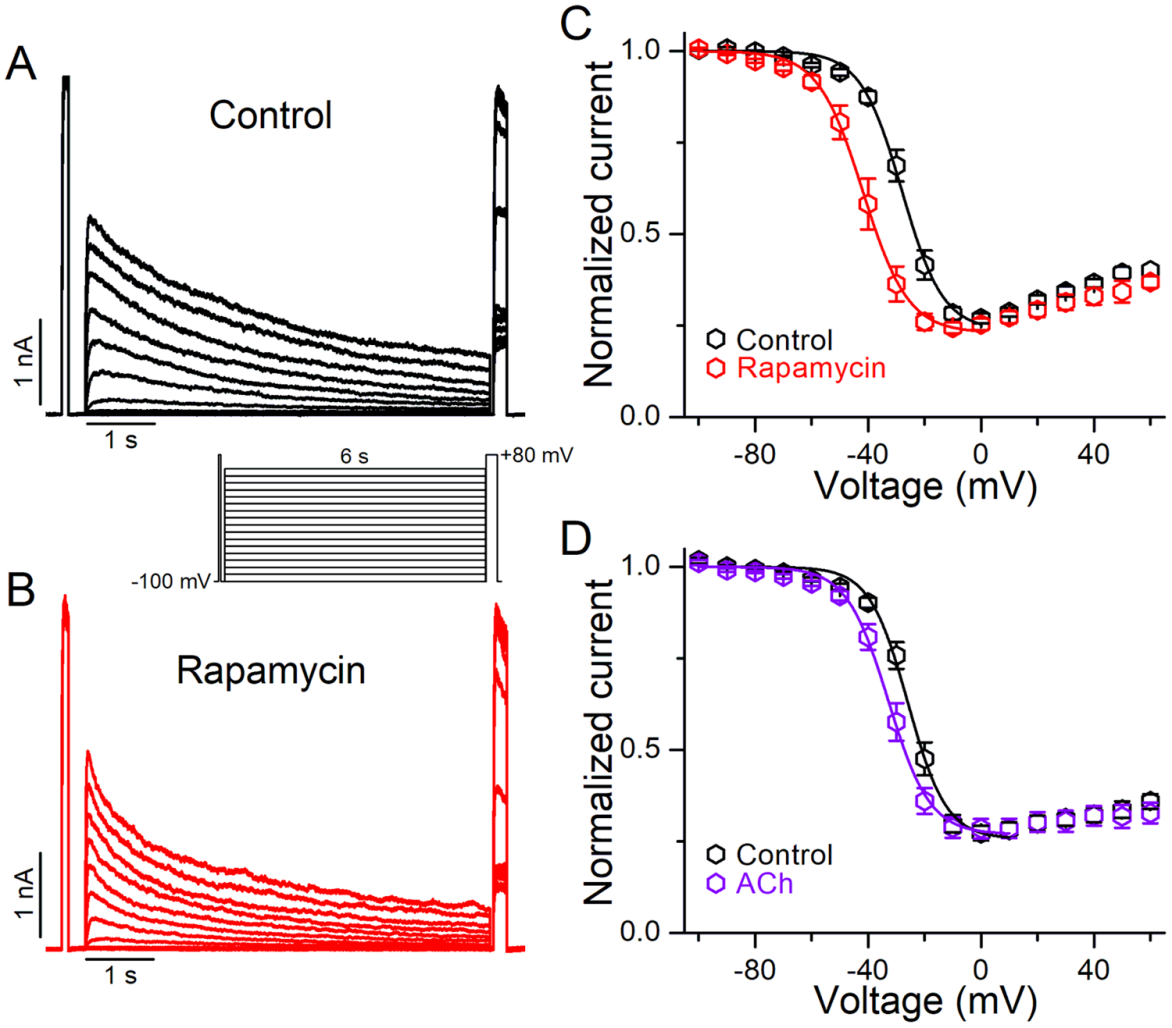
Effect of PIP_2_ depletion on the voltage-dependence of inactivation of Kv2.1 channels. (**A,B**) Representative Kv2.1 currents traces obtained with an inactivation protocol (inset) before (**A**) and after application of 1 μM rapamycin (**B**). (**C**) Inactivation curves of Kv2.1 channels determined under control conditions (black) and after cells were perfused with 1 μM rapamycin (red, n = 8). (**D**) Inactivation curves of Kv2.1 channels determined under control conditions (black) and after cells were perfused with 10 μM acetylcholine (violet, n = 8). Data points are mean ± SEM.

We have already seen modulation of the voltage-dependence of inactivation of Kv2.1 channels in excised inside-out patches that could be prevented (at least partially) by exogenous PIP_2_ (Fig. 2C). Therefore, we looked next for changes in the V_1/2_ of inactivation after PIP_2_ depletion in intact cells. Figure 5A,B shows representative current traces before (control, A) and after application of rapamycin (B), recorded with the protocol shown in the inset. After PIP_2_ depletion by rapamycin application, the V_1/2_ of inactivation was shifted toward more negative voltages relative to the control (Fig. 5C). The V_1/2_ in the control was −28.3 ± 1.4 mV (*k* = 5.6 ± 0.3 mV), while it was −41.3 ± 2.0 mV (*k* = 5.6 ± 0.2 mV) following rapamycin application (i.e. a 13 mV leftward-shift, *p* < 0.001). Activation of M1R also resulted in a ~8 mV shift of the inactivation curve to hyperpolarized potentials (Fig. 5D, Supplementary Fig. S5). The V_1/2_ in the control was −24.3 ± 0.5 mV (*k* = 6.3 ± 0.3 mV), while it was −31.9 ± 1.3 mV (*k* = 6.6 ± 0.4 mV) following M1R activation (*p* < 0.001). These results also demonstrate modulation of the inactivation gating of Kv2.1 channels by PIP_2_ in whole cells.

### Closed-state inactivation in Kv2.1 is more favorable following PIP_2_ depletion

**Figure 6:**
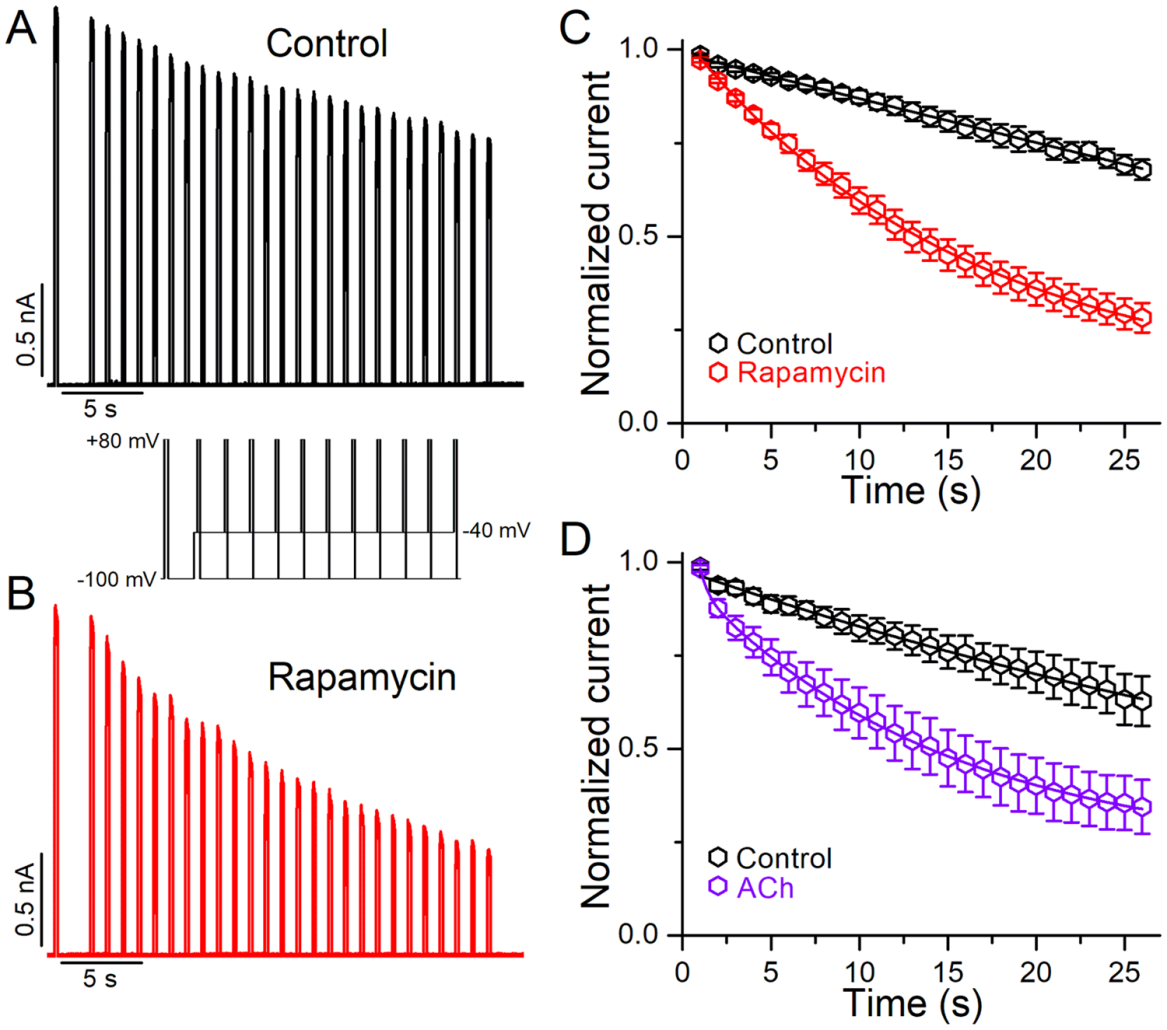
Effect of PIP_2_ depletion on the kinetics of Kv2.1 closed-state inactivation. (**A,B**) Representative Kv2.1 currents traces obtained with a closed-state inactivation protocol (inset) before (**A**) and after application of 1 μM rapamycin (**B**). (**C**) Temporal course of closed-state inactivation determined in control conditions (black) and after cells were perfused with 1 μM rapamycin (red, n = 9). (**D**) Temporal course of closed-state inactivation determined in control conditions (black) and after cells were perfused with 10 μM acetylcholine (violet, n = 7). The data points represent the current evoked by the second depolarizing pulse to + 80 mv, relative to the amplitude of the initial depolarizing pulse, plotted against the duration of the conditioning pulse at −40 mV. Data points are mean ± SEM.

An important pathway of inactivation in Kv2.1 channels originates from the closed states^20^. Because the V_1/2_ of inactivation is altered by PIP_2_ depletion, is conceivable that the inactivation of Kv2.1 channels is also modified from the closed-states. To test this hypothesis, we isolated closed-state inactivation by examining the development of inactivation at a non-activating voltage (−40 mV), before (control) and after PIP_2_ depletion by rapamycin (Fig. 6). In these experiments, a pulse to + 80 mV tested the available current after the pre-pulse to −40 mV. As the pre-pulse duration increased, channels inactivated and the current gradually decreased. Figure 6A,B shows representative currents traces before (control, A) and after application of rapamycin (B), recorded with the protocol shown in the inset. The development of closed-state inactivation was significantly accelerated after PIP_2_ depletion and well described, assuming an exponential decay (Fig. 6C). For instance, under control conditions, the time constant of the closed-state inactivation was 29.5 ± 6.8 s, whereas it was 13.4 ± 1.1 s following rapamycin application (*p* < 0.01). The closed-state inactivation was also accelerated by the activation of M1R (Fig. 6D, Supplementary Fig. S6), the time constant of closed-state inactivation was 32.5 ± 3.9 s, whereas it was 13.6 ± 1.9 s following M1R activation (*p* < 0.01).

### The recovery of Kv2.1 channels from inactivation is delayed by PIP_2_ depletion

**Figure 7:**
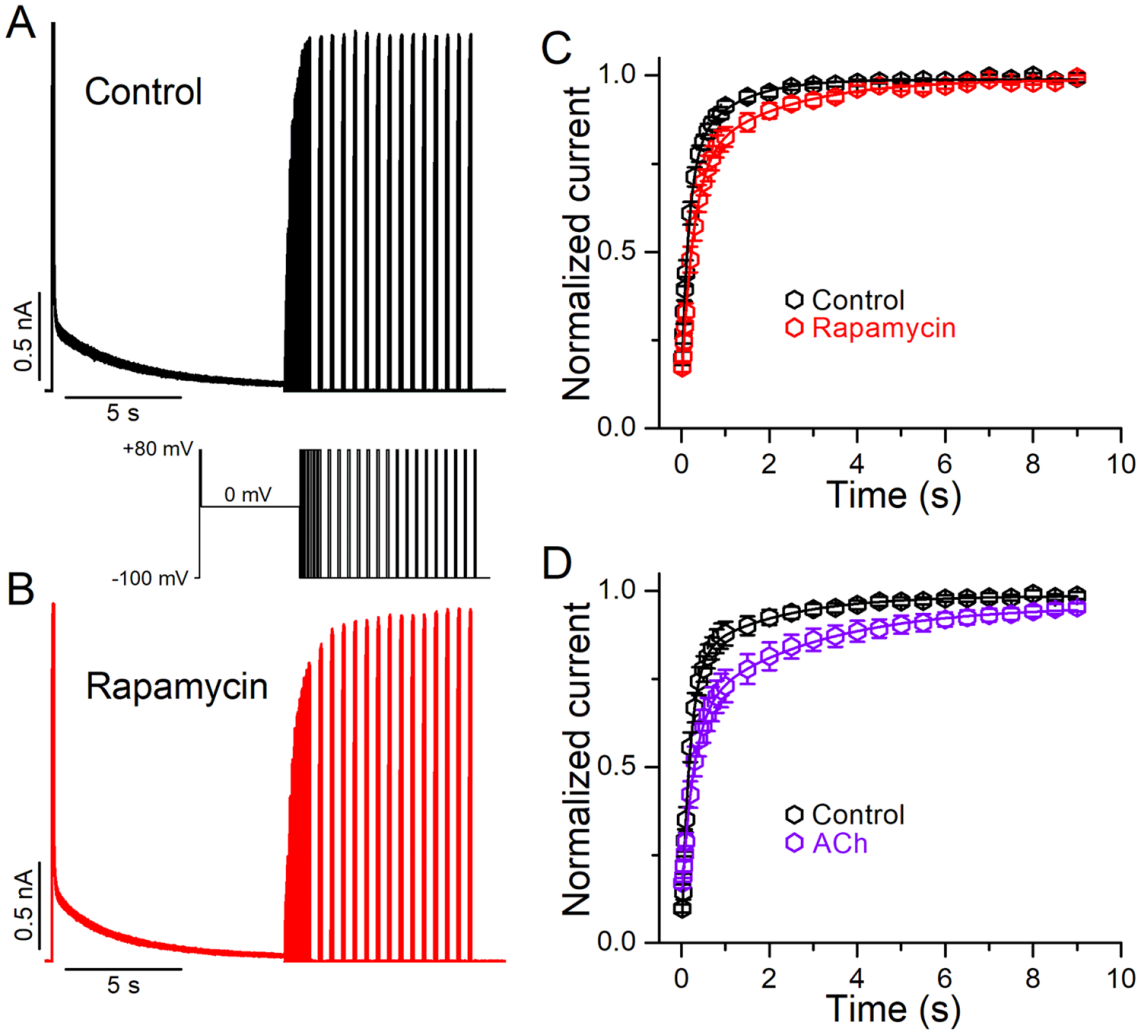
Effect of PIP_2_ depletion on the recovery kinetics of Kv2.1 channels from inactivation. (**A,B**), Representative Kv2.1 current traces recorded with a 3-pulse protocol (inset) before (**A**) and after application of 1 μM rapamycin (**B**). (**C**) Temporal course of recovery from inactivation determined in control conditions (black) and after cells were perfused with 1 μM rapamycin (red, n = 11). (**D**) Temporal course of recovery from inactivation determined in control conditions (black) and after cells were perfused with 10 μM acetylcholine (violet, n = 8). The data points represent the peak current evoked by the third pulse (P3), normalized to the peak current elicited by the identical first pulse (P1) and plotted as a function of the time interval at the holding potential (−100 mV). Data points are mean ± SEM.

Next, we examined the time course of the recovery from inactivation of Kv2.1 channels. Recovery from inactivation was measured using the protocol shown in the inset of Fig. 7. Figure 7A,B shows representative current traces before (control, A) and after application of rapamycin (B). The recovery kinetics of Kv2.1 current were best fit by a double exponential function that was modestly but significantly altered by PIP_2_ depletion (Fig. 7C). The two recovery time constants were 0.15 ± 0.02 s and 1.07 ± 0.6 s for control, and 0.3 ± 0.03 s and 2.1 ± 0.5 s following rapamycin application (*p* < 0.01). Similar effects on the recovery kinetics were observed after M1R activation (Fig. 7D, Supplementary Fig. S7), The two recovery time constants were 0.18 ± 0.05 s and 1.9 ± 0.35 s for control, and 0.32 ± 0.5 s and 4.1 ± 0.5 s following M1R activation (*p* < 0.01).

### Allosteric model of the effects of PIP_2_ depletion on Kv2.1 channels

**Figure 8:**
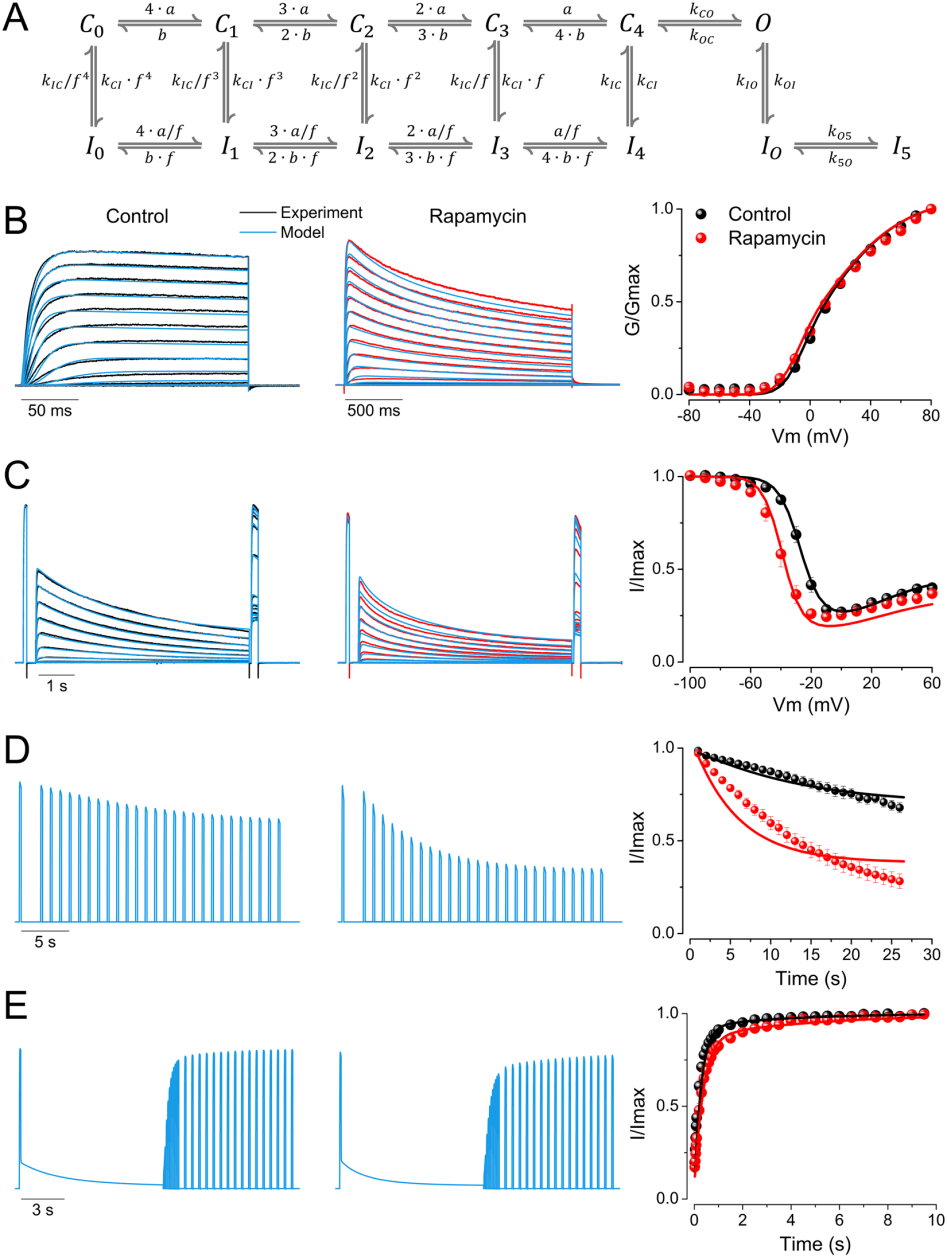
Kinetic model and simulated Kv2.1 currents. (**A**) Kinetic model:$\,{C}_{i}$,$\,{I}_{i}$, and $O$ represent closed, inactivated and opened states, respectively. Rate constants $a$, $b$ and ${k}_{OC}$ depend exponentially on membrane voltage, and ${k}_{CO}$, ${k}_{IC}$, ${k}_{CI}$, ${k}_{IO}$, ${k}_{OI}$, ${k}_{O5}$, and ${k}_{5O}$ are voltage independent rate constants (Table 1). Voltage-dependent activation and inactivation from closed states are coupled by an allosteric factor $\,f$. (**B-E**), Simulated currents (blue) reproduced from the kinetic model shown in (**A**), in control conditions (left panel) and rapamycin conditions (middle panel), using values from Table 1, and by applying the corresponding voltage protocols shown in Figs 3–6 (insets). Experimentally obtained currents are shown in black (control) and red (rapamycin). Curves plotted in right panels (continuous lines) were obtained from simulated currents in control (black) and rapamycin (red) conditions. Symbols represent the experimental data.

To better understand the mechanism by which PIP_2_ alters the function of Kv2.1 channels, we constructed a kinetic model. Figure 8A shows a 13-state model used to reproduce the effects of PIP_2_ depletion induced by rapamycin on Kv2.1 channels. As previously proposed by others^19,20^, in the building of a Kv2.1 channel model we assumed a sequential activation of the four channel subunits in the closed state (${C}_{0}$, ${C}_{1}$, ${C}_{2}$, ${C}_{3}$, and ${C}_{4}$), before the channel could reach the open state (O). Then, from either the open or closed states the channel could inactivate (${I}_{0}$, ${I}_{1}$, ${I}_{2}$, ${I}_{3}$, ${I}_{4}$, ${I}_{o0}$, ${I}_{5}$). Transitions between states were controlled by voltage dependent exponential rate constants. At a holding potential of −100 mV, channels dwelled predominantly in the ${C}_{0}$ state, and went to constitutive closed states (${C}_{1}$, ${C}_{2}$, ${C}_{3}$ to ${C}_{4}$) as the membrane voltage depolarized until the channels reached the open state (O). At the same time, as the membrane was depolarized the Kv2.1 channel could reach the inactivated states from the closed states. The model contains 11 free parameters (Table 1) whose values need to be determined from experimental data. To obtain the values of the parameters of each rate constant, we performed best global fits using ICHMASCOT^23^. We constrained the calculation by including in the fitting procedure the following data set: (a) the time course of the potassium currents at different membrane voltages (Fig. 4A,B), (b) the time course of currents induced by long pulses at different inactivating voltages (Fig. 5A,B), (c) the time course of inactivation from closed-states (Fig. 6A,B), and (d) the time course of recovery from inactivated states (Fig. 7A,B). Figure 8B–E (left panel) shows in blue color examples of best fits of potassium currents (black traces) obtained from untreated cells. In general, the model fits the Kv2.1 channel properties well, under control conditions. To fit potassium currents evoked in the presence of rapamycin, we restricted the number of free parameters assuming that rate constants, ${k}_{CO}\,\,$and ${k}_{OC}$, which mediate the closed-open and open-closed transitions, respectively, did not change. We based this assumption on the observation that the kinetic constants of activation and deactivation as a function of membrane voltage were not statistically different between control and rapamycin conditions. Additionally, the voltage-dependence of the conductance, measured at maximum current, was equal in both conditions (Fig. 4C). On the other hand, the ability of Kv2.1 channels to reach the inactivated state when PIP_2_ was depleted, from the open (Fig. 5) or the closed-state (Fig. 6), suggest that $f$, ${k}_{CI}$, ${k}_{OI}$, and ${k}_{O5}$ are modified. At the same time, the data presented in Fig. 7 show that channel current could be recovered almost back to control levels. Because of this reason $\,b$, ${k}_{IC}$, ${k}_{IO}$, and ${k}_{5O}$ were free to compensate for the more pronounced inactivation observed in the presence of rapamycin. The modified kinetic model fits the Kv2.1 channel once PIP_2_ is depleted by rapamycin. Figure 8B–E (middle panel) shows the best fits in blue on top of experimental recordings (red traces). The best-fit parameter values for the control and rapamycin conditions obtained this way are listed in Table 1.

**Table 1:** Values of the rate constants parameters for kinetic model of Kv2.1.

Parameter	Control condition	Rapamycin condition
${{a}}_{{o}}\,({{s}}^{-1})$	76.73	76.73
${{z}}_{{a}}$	1.31	1.31
${{b}}_{{o}}\,({{s}}^{-1})$	5.21	1.58
${{z}}_{{b}}$	−0.89	−1.16
${{k}}_{{o}}\,({{s}}^{-1})$	145.82	145.82
${{z}}_{{k}}$	−0.96	−0.96
${{k}}_{{CO}}\,({{s}}^{-1})$	95.66	95.66
${{k}}_{{CI}}$ $({{s}}^{-1})$	0.45	0.61
${{k}}_{{IC}}\,({{s}}^{-1})$	0.016	0.033
*f*	0.251	0.327
${{k}}_{{OI}}\,({{s}}^{-1})$	0.34	0.96
${{k}}_{{IO}}\,({{s}}^{-1})$	1.56	1.50
${{I}}_{05}\,({{s}}^{-1})$	2.91	0.80
${{I}}_{50}\,({{s}}^{-1})$	0.78	0.31

$a$ $(={a}_{o}\exp ({z}_{a}\frac{VF}{RT}))$ $b$ $(={b}_{o}\exp ({z}_{b}\frac{VF}{RT}))$, and ${k}_{OC}$ $(={k}_{o}\exp ({z}_{k}\frac{VF}{RT}))$ are voltage dependent rate constants: ${a}_{o}$, ${b}_{o}$, and ${k}_{o}$ are the rates at V = 0 mV, ${z}_{a}$, ${z}_{b}$, and ${z}_{k}$ are the corresponding equivalent electronic charges, V is the voltage, F is the Faraday constant, R is the gas constant, and T is the absolute temperature.

To make sure that the model captured steady-state properties of Kv2.1 channels, we simulated the currents using IonChannelLab^24^. The sets of rate constants are listed on Table 1. The simulated currents were then used to determine the voltage-dependent activation, voltage-dependent inactivation, time course of inactivation and time course of recovery from inactivation. In Fig. 8B–E (right panel), we compared the experimentally determined (black dots = control; red dots = rapamycin) voltage-dependence of activation (B), voltage-dependence of inactivation (C), development of closed-state inactivation (D), and recovery from inactivation (E), to those predicted by the model (continuous black and red lines). In general, the model nicely predicted most of these properties. However, the development of closed-state inactivation (Fig. 8D, right panel) was faster than the experimental data; the experimental inactivation time constant was 29.5 ± 6.8 s vs 14.4 s obtained from the simulated currents. This discrepancy was larger for the rapamycin condition. The model predicts a time constant that is about ~2.7 times faster than that calculated experimentally. These discrepancies may be due to new inactivated states that the channel could occupy when PIP_2_ becomes depleted. Additional studies will be necessary to resolve these differences.

## Discussion

Numerous ion channels have been reported to be PIP_2_ sensitive^15^. However, differences between intact cells and excised patches have been observed regarding the modulation of Kv channels by this lipid^13,14^. Kv2.1 channels were originally reported as PIP_2_ sensitive, as PIP_2_ was able to rescue Kv2.1 currents from rundown in excised patches^17^. However, later reports showed that native PIP_2_ depletion in intact cells barely affected Kv2.1 function^13^. Here, we have further investigated the regulation of Kv2.1 channels by PIP_2_ using different approaches. We found that inactivation of Kv2.1 channels is sensitive to PIP_2_ levels.

First, we confirmed that Kv2.1 currents can be rescued from rundown by application of exogenous PIP_2_ or Mg-ATP to excised inside-out patches (Fig. 1A–C and Supplementary Fig. S2). Additionally, we found that Kv2.1 current rundown depends on the holding potential (Fig. 2). Our data indicate that this phenomenon is related to the modulation of the inactivation of Kv2.1 in excised inside-out patches. After patch excision, the inactivation curves shift to hyperpolarized potentials (Δ48.6 mV compared to whole-cell, Fig. 2C), and hence, at −80 mV (the holding potential of most of our experiments) the channels can inactivate, leading to a gradual loss of Kv2.1 currents. This modulation of inactivation under inside-out conditions was previously reported for Kv4 channels^19^, channels which, like Kv2.1, possess preferential closed-state inactivation. The fact that PIP_2_ can recover Kv2.1 currents from rundown is due to the fact that it can restore (at least partially) the changes in inactivation that occurred following patch excision (Fig. 2C).

It has been proposed that the application of exogenous PIP_2_ to the cytoplasmic face of excised patches could produce physiologically irrelevant results. Consequently, it has been suggested that the use of tools to deplete PIP_2_ that maintain the cellular environment as stable as possible would produce more reliable data^13,14^. Considering these potential drawbacks, we turned to strategies to deplete native PIP_2_ in whole-cell experiments, to test whether we would corroborate our results from inside-out patches.

Depletion of PIP_2_ on whole-cell experiments by means of the recruitment of a lipid 5-phosphatase (FKBP-Inp54p) to the plasma membrane, or the activation of a Gq coupled receptor (M1R) with the consequent activation of phospholipase C (PLC) and PIP_2_ hydrolysis, confirmed the modulation of the inactivation gating of Kv2.1 channels by PIP_2_.

Kv2.1 channels exhibited their characteristic U-shaped voltage-dependence of inactivation (Fig. 5C,D). It has been suggested that U-shaped inactivation consists of a mix of both open- and closed-state inactivation with preferential inactivation from closed states throughout the voltage range^20^. PIP_2_ depletion did not modify the U-shaped inactivation curve, suggesting that preferential inactivation from closed-states was conserved (Fig. 5C,D); in fact, the inactivation from closed-states was favored after PIP_2_ depletion (Fig. 6). In agreement with a more favorable inactivation from closed-states after PIP_2_ depletion, the inactivation curve was shifted toward negative voltages (Fig. 5C,D). Furthermore, the recovery of Kv2.1 channels from inactivation was delayed after PIP_2_ depletion (Fig. 7), indicating that all inactivation parameters are modulated by PIP_2_. Interestingly, the changes in the inactivation gating occurred in the absence of a significant alteration of the activation mechanism (Figs 4, 8), suggesting that both mechanisms are uncoupled in Kv2.1 channels, contrary to what has been suggested for other Kv channels^25^.

To understand the modulation of Kv2.1 inactivation after PIP_2_ depletion, we incorporated our experimental results into a kinetic model (Fig. 8). Quantitatively, there is strong voltage-dependence between closed-state transitions (Table 1): 2.2 ${e}_{0}$ and 2.47 ${e}_{0}$ with and without PIP_2_, respectively, where ${e}_{0}$ is the electronic charge, while in the opening transition the voltage sensitivity is 0.96 ${e}_{0}$. Therefore, to achieve the open state (O), the total equivalent electronic charges are 9.7 ${e}_{0}$ in the control condition and 10.84 ${e}_{0}$ in the rapamycin condition. We observe from ${\rm{\Delta }}{G}_{k,ctrl}-{\rm{\Delta }}{G}_{k,rap}=\,-RT\,\mathrm{ln}({k}_{ctrl}/{k}_{rap})$, where ${\rm{\Delta }}{G}_{k,ctrl}$, ${k}_{ctrl}$ and ${\rm{\Delta }}{G}_{k,rap}$, ${k}_{rap}$ are energy barriers and rate constants in the control and rapamycin conditions, respectively, that the increment of the rate constants toward inactivated states, once PIP_2_ is dephosphorylated, decreases the energy barrier ($0.3\,RT$ for ${k}_{CI:ctrl}/{k}_{CI:rap}$ and $1.0\,RT$ for ${k}_{OI:ctrl}/{k}_{IO:rap}$), hence transitions toward inactivated states are more likely. For example, at −40 mV, in control conditions, after visiting the closed states, the channel reaches the ${C}_{4}$ state with 0.01 of occupation probability after a 6-s depolarization (Fig. 8C). In contrast, the ${C}_{4}$ state has 0.03 of occupation probability when rapamycin was applied. However, the occupation probability of ${I}_{4}$ changed from 0.07 to 0.26 by applying rapamycin. High probability of occupancy of the ${I}_{4}$ state is responsible for the time acceleration from the closed state (Fig. 8D). By closing the channel from this conformational state, by applying −100 mV, and reopening with + 80 mV, Kv2.1 has 0.79 of probability of opening in the control condition, but only 0.53 in the rapamycin condition: the final result is the change of the I-V inactivation curve to negative voltages (Fig. 8C, right panel). By applying + 40 mV, a membrane voltage that favors the opening of Kv2.1, the probability of occupation of the O, ${I}_{O0}$, ${I}_{5,}$ states is 0.25, 0.06 and 0.25, respectively, under control conditions. In the presence of rapamycin these states showed a 0.18, 0.12 and 0.31 probability of occupancy. In summary, PIP_2_ depletion increases the transitions from open- and closed-states to the inactivated states, with the ${C}_{4}$ to ${I}_{4}$ being the most favorable following PIP_2_ depletion.

What are the physiological implications of our findings? Kv2.1 channels are expressed in a wide variety of cells, such as central neurons^26^, cardiomyocytes^27^, and pancreatic β cells^28^, contributing to a wide range of physiological processes. For example, Kv2.1 is involved in the glucose-stimulated insulin secretion (GSIS) in pancreatic β-cells^29^. Opening of Kv2.1 channels repolarizes β-cell action potentials, limiting Ca^2+^ entry and, thus, insulin secretion^30,31^. Interestingly, the covalent attachment of small ubiquitin-like modifiers proteins to Kv2.1 channels (SUMOylation) modulates pancreatic β-cell excitability. SUMOylation modulates the inactivation gating of Kv2.1, which resulted in the widening of β-cell action potential and a decrease in firing frequency^4,5^, leading to an increase in the glucose-dependent insulin secretion. In this context, the modulation of Kv2.1 channels inactivation by PIP_2_ could result in similar effects. For instance, PIP_2_ levels in β-cells are dynamically regulated^32^. An important mechanism of such regulation is the stimulation of muscarinic receptors by acetylcholine, which is the major parasympathetic neurotransmitter released within pancreatic islets during food intake^33^. Thus, muscarinic signaling could be influencing the functioning of Kv2.1 channels, influencing β-cell excitability.

In summary, our data indicate that PIP_2_ modulates the inactivation gating of Kv2.1 channels, which could have an influence on the excitability of Kv2.1 expressing cells.

## Methods

### Reagents

Rapamycin ready-made solution (2.74 mM in DMSO) and Acetylcholine (ACh) chloride were purchased from Sigma-Aldrich (St. Louis, MO, USA). ACh was dissolved in water to make a stock solution of 25 mM. The stock solutions were diluted in bath solution to the final concentrations required for patch-clamp recordings. L-α-phosphatidylinositol-4,5-bisphosphate (PIP_2_, natural from porcine brain) was purchased from Avanti Polar Lipids, (Alabaster, AL, USA) and prepared as previously described^34^.

### Cell culture and transfection

Human embryonic kidney 293 [HEK-293] (ATCC^®^ CRL-1573^TM^) cells were grown in 60-mm tissue culture dishes (Corning, Corning, NY, USA) in Dulbecco’s modified Eagle medium (DMEM, GIBCO-Invitrogen, Grand Island, NY, USA) supplemented with 10% fetal bovine serum (Corning Life Sciences, Manassas, VA, USA) and 1% Antibiotic Antimycotic solution (Sigma-Aldrich) in a humidifier incubator at 37 °C (5% CO_2_). HEK-293 cells were transiently transfected with cDNAs encoding rKv2.1 subcloned in the pXoom vector, LDR (Lyn11-targeted FRB)-CFP and FKBP (FK506-binding protein)-Inp54p (kindly provided by Dr. Bertil Hille, University of Washington, USA), and the human M1 muscarinic receptor (M1R, kindly provided by Dr. José Sánchez-Chapula, Universidad de Colima, México) with the use of Lipofectamine 2000 reagent (Invitrogen, Carlsbad, CA, USA) according to the manufacturer´s specifications. As a marker for successfully transfected cells, cDNA encoding the enhanced green fluorescent protein (EGFP) was co-transfected with the cDNAs of interest.

### Electrophysiological recordings

Electrophysiological recordings were performed at room temperature (22–25 °C) 24 h after transfection, by using the patch-clamp technique in the whole-cell and inside-out configurations. Micropipettes were pulled from borosilicate glass capillary tubes (World Precision Instruments, Sarasota, FL, USA) on a programmable puller (Sutter Instruments, Novato, CA, USA) and had resistances of 1.5–2.5 MΩ when filled with the internal solution. Currents were recorded using an Axopatch 200B amplifier (Molecular Devices, Sunnyvale, CA, USA). Data acquisition and generation of voltage-clamp protocols were performed using a Digidata 1440 A interface (Molecular Devices) controlled by the pCLAMP 10 software (Molecular Devices). For whole-cell recordings, the external solution contained (in mM): 135 NaCl, 4 KCl, 1 MgCl_2_, 10 HEPES, 1.8 CaCl_2_, and 10 glucose (pH was adjusted to 7.4 with NaOH). The pipette solution contained (in mM): 110 KCl, 5 MgCl_2_, 5 K_4_BAPTA, 5 K_2_ATP, and 10 HEPES (pH adjusted to 7.2 with KOH). Inside-out recordings were performed using symmetrical potassium concentrations (in mM): 120 KCl, 5 K_2_EDTA, 7 KH_2_PO_4_, and 8 K_2_HPO_4_ (pH was adjusted to 7.4 with KOH). Solutions were applied using a Fast-Step Perfusion System (VC-77SP Warner Instruments, Hamden, CT, USA).

### Data analysis

Patch-clamp data were processed using Clampfit 10 (Molecular Devices) and analyzed in Origin 8.6 (OriginLab Corp. Northampton, MA, USA).

Conductance (G) was calculated using the following equation:

1 $$G=Ip/(V-{V}_{rev}),$$

where *I*_*p*_ is the peak current amplitude at the test potential *V*, and *V*_*rev*_ is the potassium reversal potential. Normalized conductance was then plotted against V to determine the voltage dependence of activation. The activation curves were then analyzed using the Boltzmann equation:

2 $$y=1/\{1+exp[-(V-{V}_{1/2})/K]\},$$

where *V* represents the test potential, *V*_*1/2*_ is the potential at which the conductance was half-maximally activated, and *K* is the slope.

The voltage dependence of Kv2.1 channel steady-state inactivation was determined using a three-step protocol. From a holding potential of −120 mV, a 100 ms depolarizing step to + 80 mV was applied (P_1_), after a brief repolarization to the holding potential, a 6-s conditioning pulse to potentials between −120 and + 80 mV was applied (P_2_) followed by a final pulse to + 80 mV (P_3_**)**. The normalized current was calculated dividing the P_3_/P_1_ current and plotted versus the conditioning potential (P_2_). The data were fitted with the Boltzmann equation:

3 $$y=1/[1+\exp (V-\,{V}_{1/2})/K],$$

Where *V* is the conditional potential, *V*_*1/2*_ is the potential at which the conductance was half-inactivated, and *K* is the slope.

Data are presented as mean ± S.E.M. (n = number of cells, recorded from at least 5 different experiments). Statistical comparisons were made using paired or unpaired Student´s t-test where applicable. Statistical significance was set at p < 0.05.

### Data availability statement

The datasets generated during and/or analysed during the current study are available from the corresponding authors upon request.

## Electronic supplementary material

Supplemetary information
