# Supplementary material for: Regulation of Kv2.1 channel inactivation by phosphatidylinositol 4,5-bisphosphate
Source: Sci Rep. 2018 Jan 29;8:1769. doi: 10.1038/s41598-018-20280-w (PMC5788980; doi:10.1038/s41598-018-20280-w)
Supplement: Supplementary file 1 — Supplemetary information [file 41598_2018_20280_MOESM1_ESM.pdf]

Supplementary material

Regulation of Kv2.1 channel inactivation by phosphatidylinositol 4,5-bisphosphate

Mayra Delgado-Ramírez[1], José J. De Jesús-Pérez[2], Iván A. Aréchiga-Figueroa[3], Jorge Arreola[2], Scott K. Adney[4,7], Carlos A. Villalba-Galea[5], Diomedes E. Logothetis[4,6*], Aldo A. Rodríguez-Menchaca[1*].

[1]Departamento de Fisiología y Biofísica, Facultad de Medicina, Universidad Autónoma de San Luis Potosí, San Luis Potosí, SLP 78210, México.

[2]Instituto de Física, Universidad Autónoma de San Luis Potosí, Universidad Autónoma de San Luis Potosí, San Luis Potosí, SLP 78290, México.

[3]CONACYT, Facultad de Medicina, Universidad Autónoma de San Luis Potosí, San Luis Potosí, SLP 78210, México.

[4]Department of Physiology and Biophysics, Virginia Commonwealth University School of Medicine, Richmond, VA 23298

[5]Department of Physiology and Pharmacology, Thomas J. Long School of Pharmacy & Health Sciences, University of the Pacific, Stockton, CA 95211, USA

[6]Department of Pharmaceutical Sciences, School of Pharmacy, Bouvé College of Health Sciences, Northeastern University, Boston, MA 02115, USA

[7]Current Address: Department of Neurology, Northwestern University, Chicago, IL 60611

[*]Corresponding authors

Diomedes E. Logothetis (email: d.logothetis@northeastern.edu)

Aldo A. Rodríguez-Menchaca (email: aldo.rodriguez@uaslp.mx)

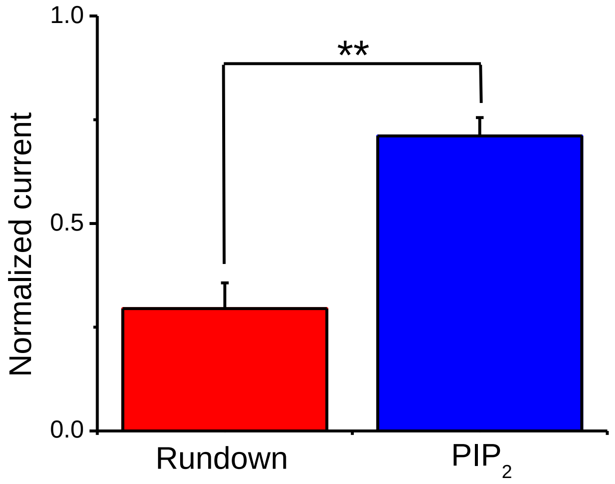

Fig. S1. Average normalized current amplitudes after rundown and exposure to 20 µM PIP_2 (n= 4, ** $p < 0.01$). Error bars represent ± SEM.

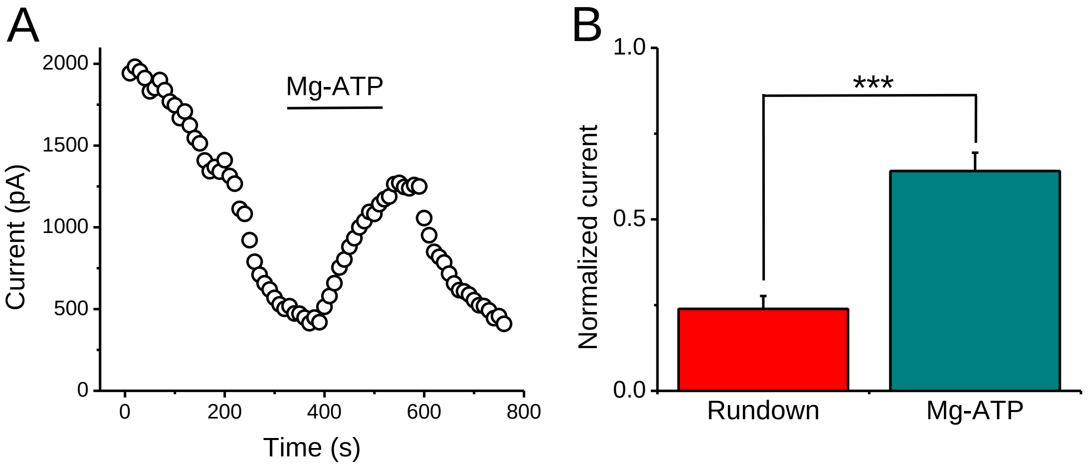

Fig. S2. Effect of 2mM Mg-ATP on the activity of Kv2.1 channels in excised inside-out patches. *A*, representative temporal course of the current amplitude at +60 mV in absence and presence of 2 mM Mg-ATP. *B*, Average normalized current amplitudes after rundown and exposure to 2 mM Mg-ATP (n= 7, *** $p < 0.001$). Error bars represent ± SEM.

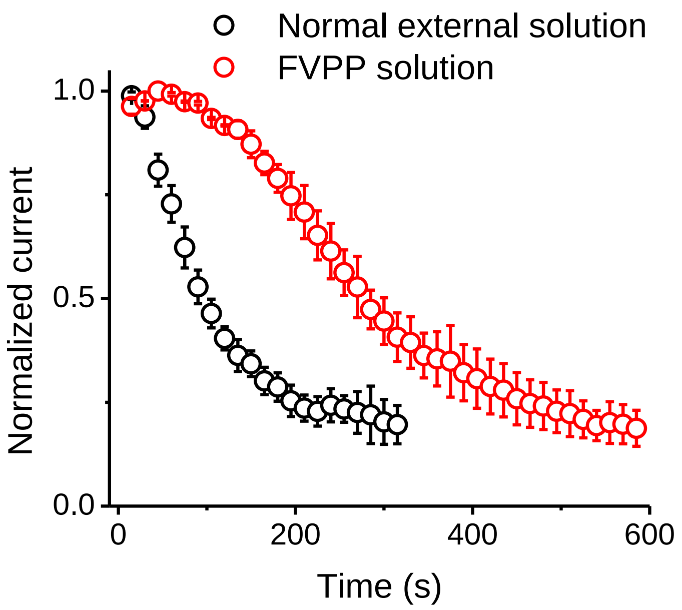

Fig. S3. Temporal course of the current amplitude at +60 mV in normal external solution (black circles) or FVPP (red circles) solutions. Current is normalized to that obtained immediately after patch excision. FVPP solution contains fluoride, vanadate and pyrophosphate to in inhibit lipid phosphatases. Data points are mean ± SEM (n= 5).

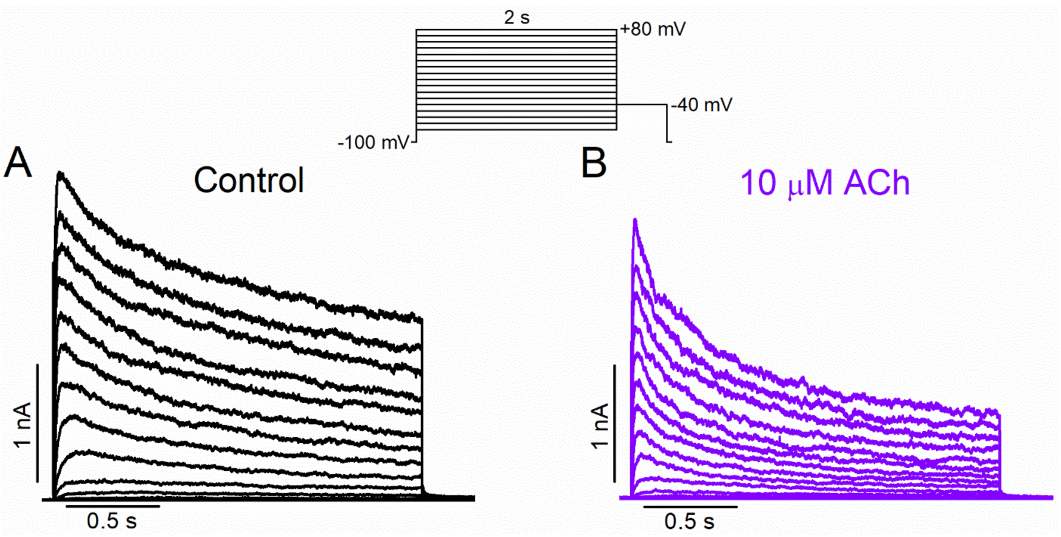

Fig. S4. Effect of M1R activation on the voltage-dependence of activation of Kv2.1 channels. *A-B*, representative Kv2.1 currents traces obtained in response to an activation protocol (*inset*) before (*A*) and after application of 10 μM acetylcholine (*B*).

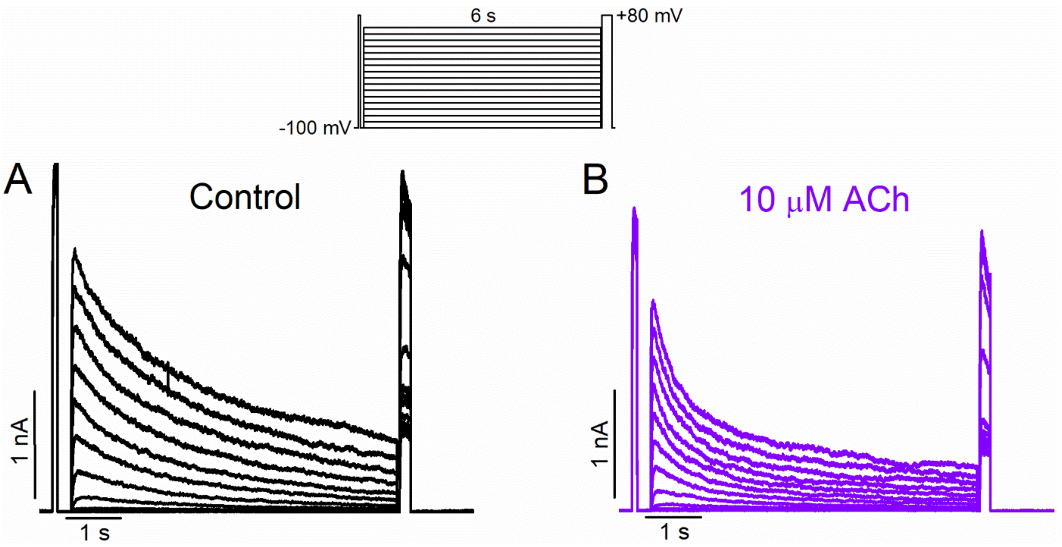

Fig. S5. Effect of M1R activation on the voltage-dependence of inactivation of Kv2.1 channels. *A-B*, representative Kv2.1 currents traces obtained with an inactivation protocol (inset) before (*A*) and after application of 10 μM acetylcholine (*B*).

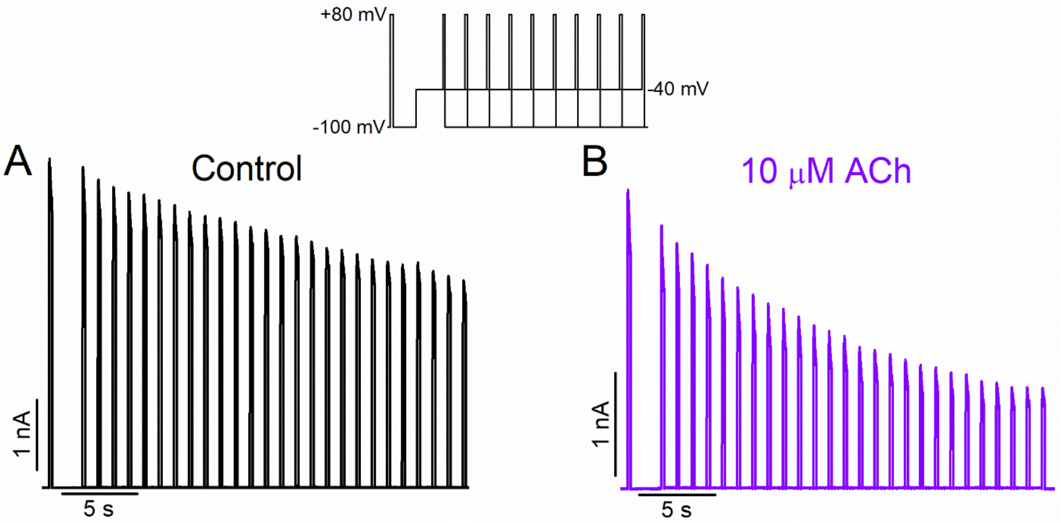

Fig. S6. Effect of M1R activation on the kinetics of Kv2.1 closed-state inactivation. *A-B***,** representative Kv2.1 currents traces obtained with a closed-state inactivation protocol (inset) before (*A*) and after application of 10 μM acetylcholine (B).

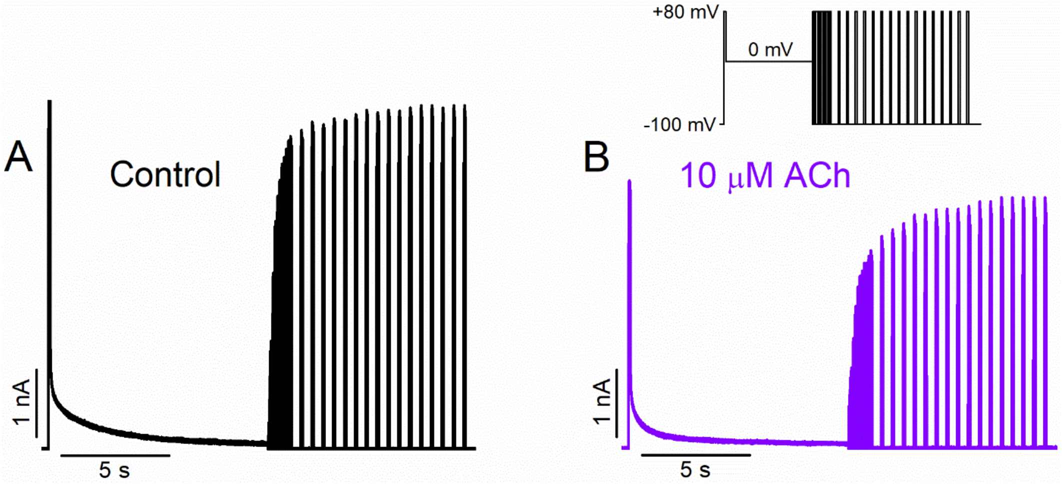

Fig. S7. Effect of M1R activation on the recovery kinetics of Kv2.1 channels from inactivation. *A*-*B*, representative Kv2.1 current traces recorded with a 3-pulse protocol (inset) before (*A*) and after application of 10 μM acetylcholine (B).
